# Innovative therapeutic strategies using ADHD medications tailored to the behavioral characteristics of patients with chronic pain

**DOI:** 10.3389/fphar.2025.1500313

**Published:** 2025-02-26

**Authors:** Satoshi Kasahara, Miwako Takahashi, Takashi Suto, Taito Morita, Hideaki Obata, Shin-Ichi Niwa

**Affiliations:** ^1^ Department of Anesthesiology and Pain Relief Center, The University of Tokyo Hospital, Tokyo, Japan; ^2^ Department of Pain Medicine, Fukushima Medical University School of Medicine, Fukushima, Japan; ^3^ Institute for Quantum Medical Science, National Institutes for Quantum Science and Technology, Chiba, Japan; ^4^ Department of Anesthesiology, Gunma University Graduate School of Medicine, Gunma, Japan; ^5^ Department of Anesthesiology, Saitama Medical Center, Saitama Medical University, Saitama, Japan; ^6^ Department of Psychiatry, Aizu Medical Center, Fukushima Medical University, Fukushima, Japan

**Keywords:** nociplastic pain, central sensitization, attention deficit hyperactivity disorder, ADHD medication, dopamine, norepinephrine, pain matrix, ergomania

## Abstract

Chronic pain affects a significant portion of adults and is linked to psychosocial issues, cognitive dysfunction, and psychiatric disorders, complicating treatment. Attention deficit hyperactivity disorder (ADHD) is increasingly recognized as a contributing factor to chronic pain, particularly nociplastic pain, with a notable prevalence of comorbidity between ADHD and conditions like fibromyalgia and chronic low back pain. ADHD behaviors such as impulsivity and overactivity can exacerbate pain by leading patients to seek risky treatments or discontinue care prematurely. ADHD medications are expected to alleviate pain severity by improving associated cognitive dysfunction and addressing central sensitization, a fundamental mechanism in chronic pain. Brain abnormalities in ADHD contribute to increased spontaneous activity in the anterior cingulate cortex-posterior insular pathway due to neuroinflammation, alterations in action potential firing, and changes in transmission pathways in the spinal dorsal horn. Additionally, increased norepinephrine synthesis and reduced transmission efficiency amplify nociceptive information from the periphery and facilitate central sensitization in ADHD. Beyond typical ADHD medications like central stimulants, norepinephrine reuptake inhibitors, and alpha-2 receptor agonists, various antidepressants, mood stabilizers, antipsychotics, Parkinson’s disease medications, and antidementia medications have proven effective in alleviating ADHD symptoms. These medications, effective for ADHD, may offer innovative solutions for managing chronic pain by targeting both the cognitive/behavioral dysfunction and central sensitization observed in chronic pain comorbid with ADHD. Further research into these mechanisms could lead to new, more effective pharmacological treatments for chronic pain with comorbid ADHD, a condition that is often overlooked.

## 1 Introduction

Chronic pain affects 13%–50% of adults (Mills et al., 2019) and is associated with psychosocial factors, cognitive dysfunction, and psychiatric disorders. Moreover, inadequate management of pain and the underlying physical conditions that cause it can also lead to cognitive impairment and psychiatric disorders. This complexity makes assessing and treating chronic pain particularly challenging and imposes an extremely high economic burden (Sarria-Santamera et al., 2022). Consequently, elucidating the pathophysiological mechanisms of chronic pain is vital for discovering new pharmacological targets.

Understanding the cognitive and behavioral characteristics of patients with chronic pain, both clinically and in daily life, alongside psychiatric disorders, is crucial for implementing effective pharmacological strategies targeting these mechanisms.

Patients with chronic pain resistant to multidisciplinary treatment often experience repeated accidents (Swanson et al., 1977), which has been linked to attention deficit hyperactivity disorder (ADHD), a neurodevelopmental disorder (Kaplan and Kaplan, 2006). Chronic pain patients frequently display three behavioral traits (Flor and Turk, 2015): (1) overconcentration on pain and susceptibility to distraction, (2) overactivity exceeding personal limits (traditionally called “ergomania”) (Vlaeyen and Morley, 2004), and (3) persistent, intense anger—features also characteristic of ADHD (American Psychiatric Association, 2013). Due to impulsivity and impatience, ADHD patients often pursue invasive, high-risk treatments (e.g., surgery, tooth extraction, opioids) for rapid relief or abandon treatments prematurely if results are not immediate, leading to “doctor shopping” (Kasahara et al., 2023d). Notably, 72.5% of chronic pain patients have comorbid ADHD, and ADHD medications can reduce the pain NRS score by 3.5 points (61.5%), indicating that ADHD comorbidity may drive cognitive and behavioral dysfunction in chronic pain (Kasahara et al., 2020).

Over 80% of adult ADHD cases go undiagnosed, especially in psychiatric settings (Ginsberg et al., 2014). Because most chronic pain patients receive care from orthopedic surgeons, rheumatologists, and pain specialists with limited ADHD expertise, comorbid ADHD often remains overlooked. Recognizing this gap could provide a breakthrough for new, behaviorally tailored pharmacotherapies.

Herein, we review clinical and basic research on the relationship between chronic pain and ADHD and explore the potential pathophysiological mechanisms involved. We also examine effective ADHD medications for chronic pain, offering insights into novel pharmacotherapeutic approaches.

## 2 Findings from clinical studies

An epidemiological study revealed an association between ADHD symptoms and work-related pain (Stickley et al., 2016). In 2017, chronic pain—previously categorized as psychogenic or somatoform—was redefined as nociplastic pain (NP), recognized alongside nociceptive and neuropathic pain (Kosek et al., 2016; Aydede and Shriver, 2018). NP is believed to result from central sensitization, involving plastic changes in central nervous system circuits that amplify nociceptive signals (Kosek et al., 2021; Treede et al., 2019; Nijs et al., 2021). NP rarely appears in isolation, instead presenting with hyperalgesia, fatigue, sensory hypersensitivity, sleep disturbances, mood disorders, or cognitive dysfunction in concentration and memory (Kosek et al., 2021), which—combined with psychosocial factors—makes treatment particularly challenging.

Recent findings indicate ADHD, a developmental disorder, frequently coexists with representative NP-related conditions such as fibromyalgia (Pallanti et al., 2021), chronic low back pain (Kasahara et al., 2021b), idiopathic orofacial pain (Kasahara et al., 2023b), chronic chest pain (Zain et al., 2023), and chronic abdominal pain (Asztély et al., 2019; Kasahara et al., 2024). ADHD may contribute to central sensitization and cognitive impairments in NP, including attention deficits and sensory hypersensitivity (Ibrahim and Hefny, 2022). Historical figures with NP, such as John F. Kennedy, who suffered from chronic low back pain (Kasahara et al., 2022a), and Margaret Mitchell, the author of *Gone with the Wind*, who struggled with fibromyalgia and experienced multiple car accidents (Kasahara et al., 2021a), were also speculated to have had comorbid ADHD.

**FIGURE 1:**
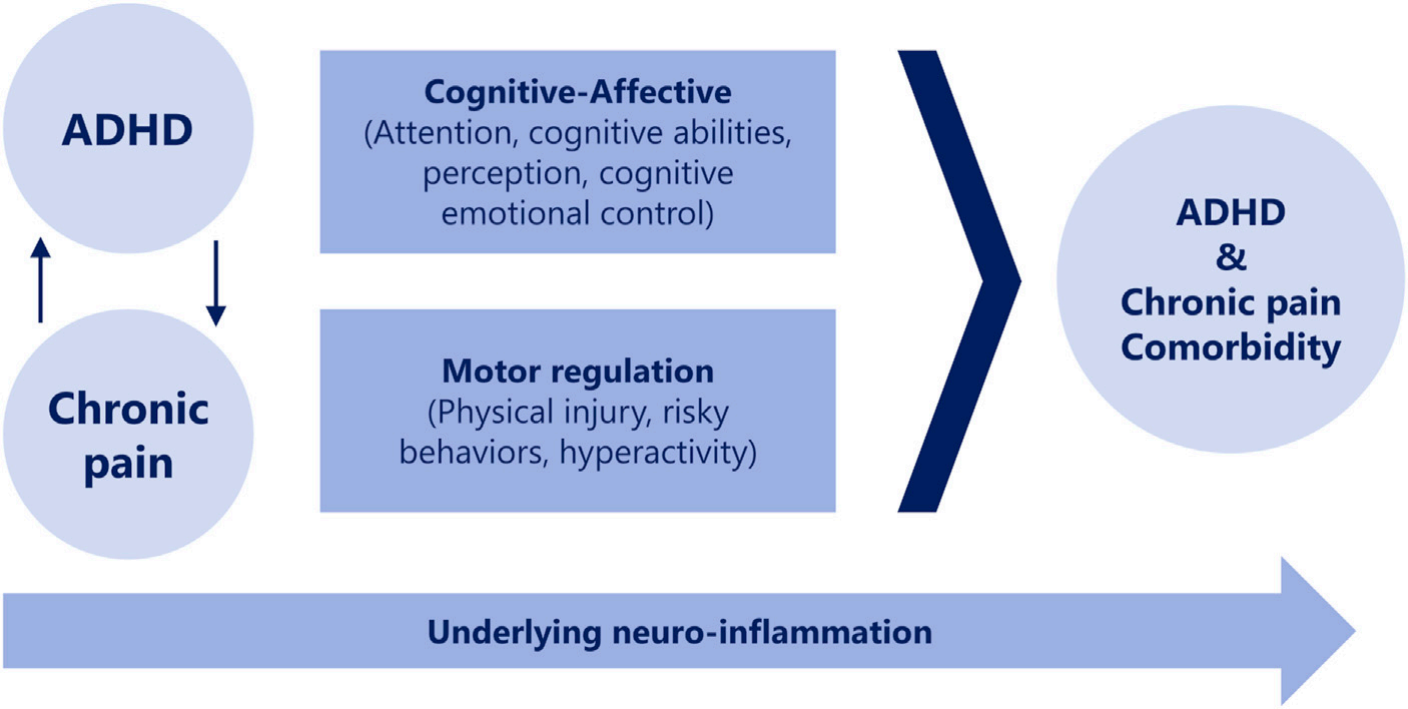
Potential associations between chronic pain and ADHD over time. (Battison et al., 2023). ADHD, attention-deficit hyperactivity disorder.

The coexistence of ADHD and chronic pain is thought to stem from dual impairments in cognitive-emotional factors and motor control associated with ADHD. ADHD exacerbates pain, and pain, in turn, worsens ADHD, with central nervous system inflammation potentially perpetuating this relationship (Battison et al., 2023) (Figure 1). Individuals with ADHD have been shown to exhibit considerably more motor control issues and increased muscle tension than those without ADHD, which are also associated with widespread and severe pain (Stray et al., 2013).

Furthermore, when ADHD accompanies NP, ADHD medications have been reported to improve pain and the associated cognitive dysfunctions. (Kasahara et al., 2017; Kasahara et al., 2022b; Kasahara et al., 2023a; Kasahara et al., 2023b; Kasahara et al., 2023c; Kasahara et al., 2023d; Kasahara et al., 2023e; Zain et al., 2023; Kasahara et al., 2024). ADHD medications administered to patients with NP have also been shown to regulate blood flow in the prefrontal cortex, precuneus, anterior cingulate cortex, and insular cortex—regions involved in the core pain matrix and related emotional processing areas (Garcia-Larrea and Peyron, 2013; Kasahara et al., 2023a; Kasahara et al., 2023c; Kasahara et al., 2023e; Kasahara et al., 2024). Notably, increased blood flow in the precuneus showed a positive correlation with NP severity scores. In the group that responded to methylphenidate, the precuneus was identified as a region of significantly elevated blood flow before treatment compared to that of after treatment. In typical cases, a decrease in precuneus hyperperfusion was accompanied by an increase in blood flow in the prefrontal cortex (Takahashi et al., 2024). Moreover, methylphenidate has been shown to alleviate motor control issues and high muscle tension in individuals with ADHD (Stray et al., 2009. See Additional Video Files 1–4.). Additionally, ADHD medications have been found to improve family relationships, which are significant psychosocial factors contributing to the maintenance and exacerbation of chronic pain (Kasahara et al., 2024). Therefore, ADHD medications are gaining attention as a new approach for treating NP, which is often difficult to manage.

However, as previously mentioned, many chronic pain patients are treated by non-psychiatric specialists such as orthopedic surgeons, rheumatologists, or pain specialists. For these physicians, diagnosing ADHD or prescribing ADHD medications is not straightforward. Therefore, when clinical behavioral characteristics of ADHD are observed, it is practical to use self-administered questionnaires such as the Adult ADHD Self-Report Scale (Kessler et al., 2005) or the Conners' Adult ADHD Rating Scale (Conners et al., 1999) for screening and to consult with a psychiatric specialist. Both scales have also been utilized in clinical research on chronic pain (Stickley et al., 2016; Kasahara et al., 2021b). Patients with coexisting ADHD often exhibit the following behavioral characteristics (Kooij and Francken, 2010): difficulty maintaining attention and concentration, which leads to omissions when responding to questionnaires; a tendency to become bored easily and frequently change jobs; procrastination on tasks requiring effort, such as returning to work or completing administrative procedures; reliance on family members for difficult tasks; disorganized speech, resulting in scattered conversations; tardiness or forgetting appointment dates; organizational difficulties, leading to overfilled bags; fidgeting with hands or feet even while seated; hyperactivity, resulting in involvement in numerous activities and subsequent exhaustion; excessive talking; impatience and impulsive pursuit of high-risk treatments, with demands such as ‘take the pain away immediately; ’ inability to control anger; over-involvement with others; and a dislike of waiting, resulting in a preference for the earliest possible appointment slots.”

## 3 Findings from basic studies

NP is associated with central sensitization (Kosek et al., 2021). Recently, basic research suggests that ADHD is more likely to cause central sensitization. At least 21 types of rodent ADHD model animals have been reported in basic research, with eight frequently cited models being identified as the most notable (Kantak K. M., 2022; Table 1).

**TABLE 1:** Rodent models of ADHD.

Rat models	ADHD features		
	Hyperactivity	Impulsivity	Inattention
**Neonatal 6-OHDA**	+	+	+
**Spontaneously Hypertensive Rat**	+	+	+
Poor performance in a 5-choice serial reaction time task		+	+
Naples High-Excitability Rat	+		+
Polychlorinated Biphenyl Exposure	+		+
Congenic Wiggling Rat	+	+	
**Prenatal Alcohol Exposure**	+	+	+
**Prenatal Nicotine Exposure**	+	+	+
**Lphn3 Knockout**	+	+	

Mouse models	Hyperactivity	Impulsivity	Inattention
Coloboma Mutant	+		
Thyroid Receptor β Mutant	+	+	+
**DAT Knockout**	+	+	
DAT Knockdown	+	+	
**NK1 Receptor Knockout**	+	+	
**Prenatal Nicotine Exposure**	+	+	+
CdK5 Dysregulation	+	+	
Git1 Knockout	+		
Lphn3 Knockout	+	+	
High Active Line	+	+	
DAT Val559 Knockin	+	+	
Neonatal 6-OHDA	+	+	+

The major models with more than 10 references are highlighted in bold. Cdk5, cyclin-dependent kinase 5; DAT, dopamine transporter; NK1, neurokinin-1; 6-OHDA, 6-hydroxydopamine (Kantak 2022).

In spontaneously hypertensive rats (SHR), an animal model for ADHD, noxious stimulus-induced analgesia (NSIA) (Tambeli et al., 2009) was weaker compared to the control group. Additionally, *in vivo* studies have shown that the concentration of norepinephrine (NE) in the dorsal horn of the spinal cord, which increases in response to nociceptive stimuli, decreases in SHR (Suto et al., 2023). This decrease suggests a weakened endogenous analgesia, including the descending inhibitory system, in ADHD.

In spinal cord slices, SHR exhibited a larger immunostaining area for NE-synthesizing enzymes in the dorsal horn than that of the control group, alongside higher extracellular NE concentrations than those of the control group. This suggests more active NE synthesis under pain-free conditions (Suto et al., 2023). Conversely, SHR exhibited a larger staining area for the norepinephrine transporter (NET) but a smaller staining area for the α2A receptor, the target receptor for NE, compared to the control group. This pattern indicates that excessive NE production in a pain-free state leads to NET overexpression and α2A receptor downregulation, resulting in diminished NE activity during NSIA and weakened descending inhibition.

Similarly, 6-hydroxydopamine (6-OHDA) mice, another ADHD model, showed shorter latency and a lower threshold for licking hind limbs in response to thermal or mechanical stimuli than those shown by controls, indicating heightened baseline nociceptive sensitivity (Bouchatta et al., 2022). In these mice, inhibitory synaptic connections in the spinal dorsal horn lamina II were similar to controls, but excitatory connections were significantly increased (Bouchatta et al., 2022). This suggests that ADHD-related anatomical changes, including increased excitatory pathways in the dorsal horn, may promote pain sensitization.

Central sensitization mechanisms have also been studied by examining the pathway from the anterior cingulate cortex (ACC) to the posterior insula (PI), known as the ACC-PI pathway (Bouchatta et al., 2022). In 6-OHDA-induced ADHD, spontaneous activity in this pathway was higher than that in controls, and ACC activity evoked by mechanical stimulation of the contralateral hind limb was stronger than that evoked in controls. This intensified activity boosted firing in second-order nociceptive neurons in the spinal dorsal horn, further lowering the pain threshold. Conversely, inhibiting the ACC-PI pathway in 6-OHDA mice more effectively suppressed dorsal horn action potentials and raised the pain threshold. Hence, ADHD-driven ACC-PI activity can both increase and reduce pain sensitivity, potentially triggering overactivity (ergomania).

Regarding the cause of elevated spontaneous ACC-PI activity, a sex-specific neuroinflammatory response to dopamine (DA) neuronal depletion in 6-OHDA mice has been reported (Meseguer-Beltrán et al., 2023). In males, DA depletion triggered inflammation only in the ACC, resulting in hyperactivity but no hyperalgesia. In females, inflammation occurred in the ACC-PI pathway, leading to hyperalgesia but not hyperactivity; reducing this inflammation alleviated hyperalgesia. These findings may explain why females experience higher rates of conditions like fibromyalgia (Queiroz, 2013) or burning mouth syndrome (Khawaja et al., 2023), as DA dysfunction in females may manifest primarily as hyperalgesia rather than hyperactivity.

In summary, in SHR and 6-OHDA mice, which have been reported to demonstrate a relationship between pain and ADHD, brain abnormalities in ADHD are thought to increase the spontaneous activity of the ACC-PI pathway owing to neuroinflammation, changes in the firing of action potentials and transmission pathways in the spinal dorsal horn, increased NE synthesis, and reduced transmission efficiency. These factors may amplify peripheral nociceptive information, increasing susceptibility to central sensitization. Furthermore, it has been shown that in SHR and 6-OHDA mice, hyperalgesia caused by central sensitization and ADHD symptoms, such as hyperactivity and inattention, can be improved by atomoxetine—a selective norepinephrine reuptake inhibitor (NRI) used as an ADHD medication (Sifeddine et al., 2023; Suto et al., 2023).

However, among the ADHD models listed in Table 1, only the SHR and 6-OHDA mice have been reported to show a relationship between pain and ADHD, while such investigations have not been conducted for other ADHD model animals. Therefore, there is room to explore the relationship between pain and ADHD in these other models as well. Additionally, ADHD is believed to result from gene-environment interactions, highlighting the need to develop models that integrate both genetic and environmental factors. This integration remains a challenge for ADHD models as a whole (Regan et al., 2022). Furthermore, since all rodent ADHD models exhibit hyperactivity or impulsivity, developing a model that specifically represents inattentive-type ADHD without hyperactivity or impulsivity remains an important task.

## 4 Discussion

Medications that are effective for treating ADHD may also be effective in treating chronic pain and its associated cognitive dysfunction mediated by central sensitization. Here, we briefly introduce the representative drug candidates (Table 2).

**TABLE 2:** Medications effective for ADHD.

Compounds	Medicinal classification	Main indications	Dosage
Methylphenidate	Psychostimulant	ADHD	20–60 mg/day
Lisdexamfetamine	Psychostimulant	ADHD	30–70 mg/day
Amphetamine	Psychostimulant	ADHD/Narcolepsy	5–40 mg/day
Modafinil	Psychostimulant	Narcolepsy	200 mg/day
Pemoline	Psychostimulant	Narcolepsy	56.25–75 mg/day
Atomoxetine	NRI	ADHD	40–100 mg/day
Reboxetine	NRI	Depression	8–10 mg/day
Guanfacine	α2 agonist	ADHD	1–6 mg/day
Clonidine	α2 agonist	Hypertension	0.2–0.6 mg/day
Bupropion	NDRI	Depression	150–450 mg/day
Duloxetine	SNRI	Depression	30–120 mg/day
Venlafaxine	SNRI	Depression	37.5–300 mg/day
Paroxetine	SSRI	Depression	20–60 mg/day
Desipramine	TCA	Depression	100–200 mg/day
Nortriptyline	TCA	Depression	75–150 mg/day
Amitriptyline	TCA	Depression	50–150 mg/day
Lithium	mood stabilizer	Bipolar Disorder	600–1200 mg/day
Lamotrigine	mood stabilizer	Bipolar Disorder	100–200 mg/day
Haloperidol	Antipsychotic	Schizophrenia	1–40 mg/day
Chlorpromazine	Antipsychotic	Schizophrenia	200–800 mg/day
Risperidone	Antipsychotic	Schizophrenia	2–8 mg/day
Aripiprazole	Antipsychotic	Schizophrenia/Bipolar disorder	2–30 mg/day
Pramipexole	Antiparkinsonism	Parkinson’s disease	1.5–4.5 mg/day
Selegiline	Antiparkinsonism	Parkinson’s disease	5–10 mg/day
Donepezil	Antidementia	Dementia	5–10 mg/day
Galantamine	Antidementia	Dementia	16–24 mg/day
Memantine	Antidementia	Dementia	20 mg/day

ADHD, attention deficit hyperactivity disorder; NDRI, norepinephrine-dopamine reuptake inhibitor; NRI, norepinephrine reuptake inhibitor; SNRI, serotonin norepinephrine reuptake inhibitor; SSRI, selective serotonin reuptake inhibitor; TCA, tricyclic antidepressant.

The main ADHD medications include the central stimulant methylphenidate, the selective NRI atomoxetine, the α2 receptor agonist guanfacine, and the central stimulant lisdexamfetamine (Stahl, 2021). There have been reports that the first three of these drugs have shown improvements in chronic pain and its associated cognitive dysfunction (Kasahara et al., 2023a; Kasahara et al., 2023e; Zain et al., 2023). However, even with these so-called ADHD medications approved by the Food and Drug Administration, more than 33% of children and over 50% of adults discontinue treatment within a year due to suboptimal efficacy or tolerance issues (Childress A., 2024). Therefore, alternative ADHD treatments that have proven effective for ADHD, as discussed below, are also considered pertinent (Buoli et al., 2016).

Other central stimulants such as amphetamines, modafinil, and pemoline are also considered effective for ADHD (Stahl, 2020). It has been suggested that the amphetamine, which John F. Kennedy used to treat back pain, may have also inadvertently improved ADHD symptoms (Kasahara et al., 2022a).

Additionally, antidepressants, such as duloxetine and venlafaxine, which are norepinephrine-serotonin reuptake inhibitors, have been reported to improve ADHD symptoms (Buoli et al., 2016) and NP (Kasahara et al., 2024). Traditional tricyclic antidepressants (TCAs), used to treat chronic pain (Feinmann and Harris, 1984), also improve ADHD (Banaschewski et al., 2004). TCAs inhibit norepinephrine reuptake. Dopamine reuptake occurs via norepinephrine transporters in the prefrontal cortex. Consequently, TCAs enhance both norepinephrine and dopamine levels in this region. Among TCAs, desipramine is considered the most effective for ADHD, whereas imipramine, nortriptyline, and amitriptyline are slightly less effective. On the other hand, clomipramine and protriptyline are deemed less effective and generally unsuitable due to intolerable side effects. The effects of TCAs on ADHD primarily affect behavioral symptoms rather than cognitive symptoms. Furthermore, it has been suggested that their effectiveness in treating chronic pain may have been due to the improvement of undiagnosed coexisting ADHD (Kasahara et al., 2023d). Other antidepressants, such as the selective serotonin reuptake inhibitor paroxetine, the NE-DA reuptake inhibitor bupropion, and the selective NRI reboxetine, are also considered effective for ADHD (Buoli et al., 2016). Regarding the role of serotonin in ADHD treatment, studies report that hyperactivity caused by high extracellular dopamine levels in 6-OHDA mice is regulated and improved by enhancing serotonergic tone (Regan et al., 2022). Furthermore, mood stabilizers such as lithium and lamotrigine are also considered effective for ADHD (Buoli et al., 2016; Sablaban and Sivananthan, 2022). These agents have been shown to improve ADHD symptoms associated with addictive behaviors, intense anger, and comorbid bipolar disorder (Buoli et al., 2016). Notably, the effectiveness of lithium in treating ADHD has been reported to be comparable to that of methylphenidate (Dorrego et al., 2002).

Haloperidol, chlorpromazine (Stahl, 2020), risperidone, and aripiprazole (Ghanizadeh, 2013; Lamberti et al., 2016) are effective treatments for ADHD. There have also been reports that risperidone (Kasahara et al., 2022b) and aripiprazole (Kasahara et al., 2011; Kasahara et al., 2023d) have improved chronic pain. Haloperidol and chlorpromazine are considered useful for managing hyperactivity and aggression in ADHD (Stahl, 2020). Risperidone and aripiprazole have been found beneficial for ADHD patients experiencing anxiety, irritability, depression, anger, or self-injurious behavior (Alsayouf, 2024). Antipsychotics are thought to ameliorate ADHD by addressing these cognitive-emotional factors. Notably, the effectiveness of risperidone in treating ADHD symptoms has been reported to be comparable to that of methylphenidate (Arabgol et al., 2015).

Additionally, although antipsychotics block dopamine D2 receptors and may initially seem counterproductive for ADHD, which is typically characterized by low dopamine levels, the “Complex DA Model” has been proposed to explain this phenomenon (Yanofski, 2010). According to this model, ADHD involves low tonic dopamine levels and high DA bursts. Stimulants act presynaptically to increase tonic dopamine, suppress DA bursts, and downregulate postsynaptic receptors. Conversely, antipsychotics like risperidone and aripiprazole function postsynaptically to increase tonic dopamine, suppress DA bursts, and upregulate postsynaptic receptors. Therefore, while stimulants and antipsychotics are each effective for ADHD when used alone, their combination proves particularly effective. This combined approach acts on both pre- and postsynaptic mechanisms, increasing tonic dopamine, suppressing DA bursts, and stabilizing postsynaptic receptor regulation. Consequently, this strategy is believed to prevent tolerance to treatment, minimize side effects due to receptor upregulation, and allow for low medication dosages.

DA agonists such as pramipexole (Kurlan et al., 2012) and the selective monoamine oxidase B inhibitor selegiline (Buoli et al., 2016; Akhondzadeh et al., 2003), commonly used to treat Parkinson’s disease, have also been found to be effective for ADHD by enhancing DA neurotransmission at the synapse. Pramipexole improves chronic low back pain (Kasahara et al., 2023c) and fibromyalgia (Holman and Myers, 2005). Clonidine, an α2 agonist like guanfacine and an antihypertensive, is considered effective for ADHD (Stahl, 2020) and has shown efficacy in treating chronic pain (Guay, 2001). Additionally, cholinesterase inhibitors such as donepezil (Doyle et al., 2006) and galantamine (Buoli et al., 2016), along with the N-methyl-D-aspartate receptor antagonist memantine (Surman et al., 2013), typically used as anti-dementia drugs, are known to activate dopamine neurotransmission and have been reported to be effective against ADHD. These medications may also potentially improve chronic pain.

The contents of this mini-review can be summarized as follows: The cognitive-emotional and motor control issues associated with ADHD mutually amplify and exacerbate both ADHD and pain. Neuroinflammation and alterations in neurotransmission within the ADHD brain contribute to central sensitization, amplifying nociceptive input and promoting pain chronicity. ADHD medications can correct the increased blood flow in the precuneus and decreased blood flow in the prefrontal cortex, thereby improving cognitive-emotional factors such as anxiety, depression, anger, and aggression, as well as motor control issues like increased muscle tension. These treatments have the potential to alleviate not only chronic pain itself but also its associated psychosocial issues. However, it is assumed that the ADHD phenotype encompasses multiple models, each responding differently to medication. Therefore, instead of limiting treatment to a single medication, it is pertinent to implement a treatment algorithm composed of multiple medications with different mechanisms of action, as discussed in this review (e.g., Kasahara et al., 2023d).

In the future, to develop innovative pharmacological treatments for chronic pain, it is considered important to discover approaches that utilize ADHD medications targeting the clinical features of ADHD coexisting with chronic pain and the pathophysiological mechanisms of central sensitization.

## Abbreviations

NP, nociplastic pain; 6-OHDA, 6-hydroxydopamine; SHR, spontaneously hypertensive rats; NSIA, noxious stimulus-induced analgesia; NE, norepinephrine; ACC, anterior cingulate cortex; PI, posterior insula; ACC-PI pathway, anterior cingulate cortex posterior insula; DA, dopamine; NRI, norepinephrine reuptake inhibitor.
